# Sound Source Localization Based on Multi-Channel Cross-Correlation Weighted Beamforming

**DOI:** 10.3390/mi13071010

**Published:** 2022-06-26

**Authors:** Mengran Liu, Junhao Hu, Qiang Zeng, Zeming Jian, Lei Nie

**Affiliations:** Hubei Key Laboratory of Modern Manufacturing Quantity Engineering, School of Mechanical Engineering, Hubei University of Technology, Wuhan 430068, China; mrliu@hbut.edu.cn (M.L.); hujunhao199802@163.com (J.H.); zengqiang199907@163.com (Q.Z.); leinie@hbut.edu.cn (L.N.)

**Keywords:** sound source localization, multi-channel cross-correlation coefficient, microphone array, beamforming

## Abstract

Beamforming and its applications in steered-response power (SRP) technology, such as steered-response power delay and sum (SRP-DAS) and steered-response power phase transform (SRP-PHAT), are widely used in sound source localization. However, their resolution and accuracy still need improvement. A novel beamforming method combining SRP and multi-channel cross-correlation coefficient (MCCC), SRP-MCCC, is proposed in this paper to improve the accuracy of direction of arrival (DOA). Directional weight (DW) is obtained by calculating the MCCC. Based on DW, suppressed the non-incoming wave direction and gained the incoming wave direction to improve the beamforming capabilities. Then, sound source localizations based on the dual linear array under different conditions were simulated. Compared with SRP-PHAT, SRP-MCCC has the advantages of high positioning accuracy, strong spatial directivity and robustness under the different signal–noise ratios (SNRs). When the SNR is −10 dB, the average positioning error of the single-frequency sound source at different coordinates decreases by 5.69%, and that of the mixed frequency sound sources at the same coordinate decreases by 5.77%. Finally, the experimental verification was carried out. The results show that the average error of SRP-MCCC has been reduced by 8.14% and the positioning accuracy has been significantly improved, which is consistent with the simulation results. This research provides a new idea for further engineering applications of sound source localization based on beamforming.

## 1. Introduction

Sound source localization techniques have a wide range of application prospects in civil and military systems, such as intelligent medical systems, security monitoring and sonar detection [1,2,3,4,5]. Existing sound source localization techniques can be divided into the subspace, time delay estimation and beamforming. The subspace approach uses the orthogonality between the signal and noise subspaces to determine the waveform direction, including multiple signal classification (MUSIC) and estimating signal parameters via rotational invariance techniques (ESPRIT) [6,7]. Their performance is heavily dependent on the covariance matrix estimation, which is influenced by the signal-to-noise ratio (SNR). Hu et al. proposed an improved MUSIC algorithm to calculate the spatial spectrum and achieve azimuth estimation [8]. Herzog et al. developed a novel EB-ESPRIT method to estimate the incoming wave direction of a sound source [9]. The direction estimation accuracy of studies [8] and [9] is higher than that of MUSIC and ESPRIT, respectively. However, the accuracy is still significantly reduced at a lower SNR. The time delay estimation method achieves the source location based on the arrival time difference [10]. In [11], sound source localization in an indoor environment is completed by using two dual-element arrays to estimate time delay. However, the time delay estimation method is susceptible to noise. When the number of array elements is increased, there is redundant information in the signals. The multi-channel cross-correlation coefficient (MCCC) method in [12] can improve the robustness of delay estimation through multi-element array signals. Beamforming is to obtain the direction of the sound source by summing weighted array signals, and is classified into frequency domain beamforming and time domain beamforming [13,14]. In frequency domain beamforming, many methods have been proposed to improve its performance [15,16]. Reference [15] improved the robustness by adding norm constraints and spatial smoothing techniques. Reference [16] proposed nested arrays to improve beamforming performance. Time domain beamforming is compared with frequency domain beamforming in [17]. The two beamforming methods have similar performance. Time domain beamforming is a natural broadband method which is suitable for single-frequency and multi-frequency signals, and it does not require repeated multiple frequency processing. In time domain beamforming, the steered-response power (SRP) is commonly used. Steered-response power delay and sum (SRP-DAS) is used for direction estimation based on microphone arrays. The steered-response power phase transform (SRP-PHAT) algorithm is an optimization of the SRP-DAS, which is easy to implement and has stronger robustness than SRP-DAS by whitening the signals [18,19]. The SRP-PHAT algorithm as time domain beamforming has been widely used in target tracking and distributed localization [20,21,22]. Salvati et al. reported the SRP-PHAT modification algorithm, which can speed up the operation [23]. However, the directivity based on SRP-PHAT is not outstanding in azimuth estimation, and the localization accuracy still needs further improvement.

Therefore, combining the advantages of MCCC and SRP, a new beamforming method (SRP-MCCC) is proposed in this paper. In this method, the wave direction weight (DW) is calculated by the MCCC, which adjusts the SRP value to enhance the directivity of microphone arrays and improve spatial resolution. In this paper, Section 2 describes the positioning principle of the proposed method. Then, the sound source localization simulation is reviewed in Section 3. Section 4 verifies the performance of the proposed method under experimental conditions. Finally, conclusions are given in Section 5.

## 2. Positioning Principle

**Figure 1:**
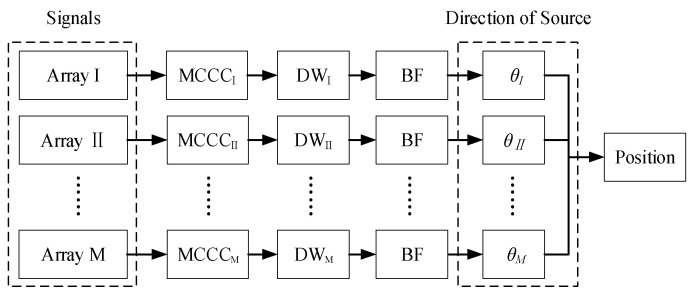
Positioning flowchart.

Figure 1 shows the calculating flow of the position. Suppose the numbers of arrays and each array elements are M and N, respectively. For each array, the MCCC is evaluated by the N-elements’ signals. The DW is constructed by using redundant information of MCCC. After obtaining the DW, the weighted beamforming is performed, and finally, the relative direction $\left(\theta_{I},{\;\theta }_{II},\;\ldots ,{\;\theta }_{M}\right)$ is found. When M≥2, the source position can be calculated from $\left(\theta_{I},{\;\theta }_{II},\;\ldots ,{\;\theta }_{M}\right)$.

### 2.1. Signal Model

**Figure 2:**
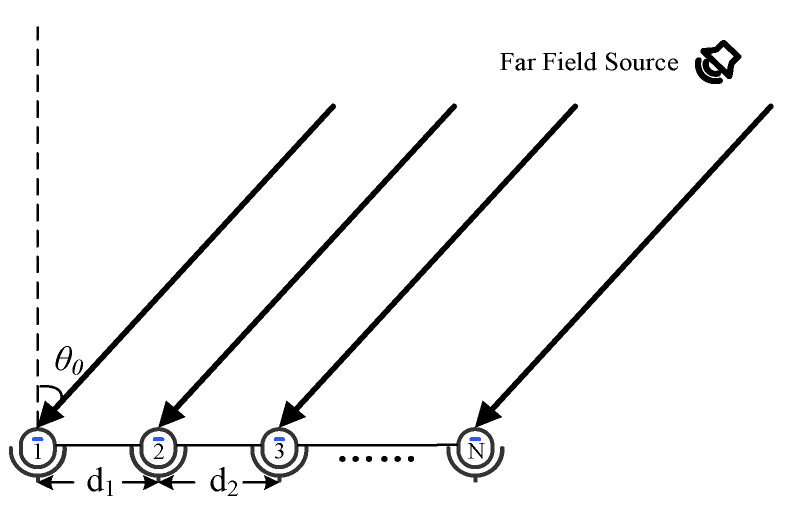
Signal and array model.

The far-field signal propagation and the N-element linear microphone array model are shown in Figure 2. $\theta_{0}$ represents the direction from the far-field source to the array, and $d_{i}$ (*i* = 1, 2……*N* − 1) denotes the spacing between the two array elements in the array.

Assume that the source signal is s(n), which is a time series. The signals received by the N-element linear microphone array can be expressed as X(n).

(1) $$X(n)=\left[\begin{array}{c} x_{1}(n)\\ x_{2}(n)\\ \ldots \\ x_{N-1}(n)\\ x_{N}(n) \end{array}\right]=\left[\begin{array}{c} \alpha_{1}s(n-t_{1})+v_{1}(n)\\ \alpha_{2}s(n-t_{2})+v_{2}(n)\\ \ldots \\ \alpha_{N-1}s(n-t_{N-1})+v_{N-1}(n)\\ \alpha_{N}s(n-t_{N})+v_{N}(n) \end{array}\right]$$

where $x_{i}\left(n\right)\left(i=1,\;2,\;\ldots ,\;N\right)$ represents the signal received by the *i*-th microphone, $\alpha_{i}$ is the attenuation coefficient of the signal received by the *i*-th microphone, $t_{i}$ is the propagation time from the sound source localization to the *i*-th microphone and $v_{i}\left(n\right)$ is the noise signal received by the *i*-th microphone.

Taking the first microphone as the reference, the aligned signal y(n, τ) can be written as:

(2) $$\begin{array}{ll} y(n,\tau ) & =[{\begin{array}{ccccc} {\alpha^{\prime }}_{1}x_{1}(n-\tau_{1}) & {\alpha^{\prime }}_{2}x_{2}(n-\tau_{2}) & \ldots & {\alpha^{\prime }}_{N-1}x_{N-1}(n-\tau_{N-1}) & {\alpha^{\prime }}_{N}x_{N}(n-\tau_{N}) \end{array}]}^{T}\\ & ={\left[\begin{array}{cccc} {\alpha^{\prime }}_{1}{s^{\prime }}_{1}(n-\tau_{1}) & {\alpha^{\prime }}_{2}{s^{\prime }}_{2}(n-\tau_{2}) & \ldots & \begin{array}{cc} {\alpha^{\prime }}_{N-1}{s^{\prime }}_{N-1}(n-\tau_{N-1}) & {\alpha^{\prime }}_{N}{s^{\prime }}_{N}(n-\tau_{N}) \end{array} \end{array}\right]}^{T}\\ & +{\left[\begin{array}{ccccc} v_{1}(n-\tau_{1}) & v_{2}(n-\tau_{2}) & \ldots & v_{N-1}(n-\tau_{N-1}) & v_{N}(n-\tau_{N}) \end{array}\right]}^{T} \end{array}$$

where ${s^{\prime }}_{i}\left(n-\tau_{i}\right)$ represents the *i*-th signal after alignment. $\tau_{i}\;\left(i=1,\;2,\;\ldots ,\;n\right)$ is the time delay from the *i*-th microphone to the first microphone, and its value is related to the position of the sound source and the microphone array structure, where $\tau_{1}=0$. ${\alpha^{\prime }}_{i}$ is the relative attenuation coefficient, and it is calculated by

(3) $${\alpha^{\prime }}_{i}=\frac{\alpha_{i}}{\alpha_{1}}\begin{array}{cc} & \left(i=1,2,\ldots ,N\right) \end{array}$$

For a linear array, the following relationship exists between $\tau_{i}$ and $d_{n}$ (the distance between the *n*-th and (*n* + 1)-th microphone):

(4) $$\tau_{i}=\{\begin{array}{c} 0\begin{array}{cc} & \left(i=1\right) \end{array}\\ \frac{\sum_{n=1}^{i-1}d_{n}}{d_{1}}\tau_{2}\begin{array}{c} \left(i\geq 2\right) \end{array} \end{array}$$

### 2.2. Direction Weight

The Pearson coefficient of the normalized signals is used to replace the value of the correlation function [24,25]. The correlation coefficient matrix of different time delays is ρ(τ):

(5) $$\begin{array}{ll} \rho (\tau ) & =Ρy(n,\tau )y{\left(n,\tau \right)}^{T}Ρ\\ & =\left[\begin{array}{cccc} \rho_{11}(\tau_{1},\tau_{1}) & \rho_{12}(\tau_{1},\tau_{2}) & \ldots & \rho_{1N}(\tau_{1},\tau_{N})\\ \rho_{21}(\tau_{2},\tau_{1}) & \rho_{22}(\tau_{2},\tau_{2}) & \ldots & \rho_{2N}(\tau_{2},\tau_{N})\\ \ldots & \ldots & \ldots & \ldots \\ \rho_{N1}(\tau_{N},\tau_{1}) & \rho_{N2}(\tau_{N},\tau_{2}) & \ldots & \rho_{NN}(\tau_{N},\tau_{N}) \end{array}\right] \end{array}$$

where

(6) $$Ρ=\left[\begin{array}{cccc} \frac{1}{\sigma_{1}} & & & \\ & \frac{1}{\sigma_{2}} & & \\ & & \ldots & \\ & & & \frac{1}{\sigma_{N}} \end{array}\right]$$

$\rho_{ij}\left(\tau \right)$ is the correlation coefficient between the *i*-th and the *j*-th alignment signal, which can be expressed as:

(7) $$\begin{array}{l} \rho_{ij}(\tau_{i},\tau_{j})=\frac{x_{i}(n-\tau_{i})\cdot x_{j}(n-\tau_{j})}{\sigma_{i}\sigma_{j}}\\ =\frac{{\alpha^{\prime }}_{i}{s^{\prime }}_{i}(n-\tau_{i})\cdot {\alpha^{\prime }}_{j}s_{j}(n-\tau_{j})}{\sigma_{i}\sigma_{j}}+\frac{{\alpha^{\prime }}_{i}{s^{\prime }}_{i}(n-\tau_{i})\cdot v_{j}(n-\tau_{j})}{\sigma_{i}\sigma_{j}}\\ +\frac{{\alpha^{\prime }}_{j}{s^{\prime }}_{j}(n-\tau_{i})\cdot v_{i}(n-\tau_{i})}{\sigma_{i}\sigma_{j}}+\frac{{v^{\prime }}_{i}(n-\tau_{i})\cdot v_{j}(n-\tau_{j})}{\sigma_{i}\sigma_{j}}\\ =r_{ij}+r_{iv_{j}}+r_{jv_{i}}+r_{v_{i}v_{j}} \end{array}$$

where $\sigma_{i}=\sum x_{i}^{2}$ is the energy of the *i*-th signal and · indicates the inner product. Observing Equation (7), it can be deduced that $r_{ij}$ is the correlation coefficient between the *i*-th signal and the *j*-th signal. $r_{{iv}_{j}}$ represents the correlation coefficient between the *i*-th signal and the *j*-th noise, and $r_{{jv}_{i}}$ is the correlation coefficient between the *j*-th signal and the *i*-th noise. $r_{v_{i}v_{j}}$ is expressed as the correlation coefficient between the *i*-th noise and the *j*-th noise. It can be seen that when ${s^{\prime }}_{i}(n-\tau_{i})={s^{\prime }}_{j}(n-\tau_{j}),\;r_{ij}$ has the maximum value. The $h_{DW}\left(\tau \right)$ is constructed by using redundant information of the MCCC. $h_{DW}\left(\tau \right)$ is calculated from the correlation coefficient matrix, which is directly related to the signal itself, and it can be represented as:

(8) $$h_{DW}(\tau )=\prod_{i=1}^{N}\prod_{j=1}^{i}\rho_{ij}(\tau_{i},\tau_{j})$$

### 2.3. Weighted Beamforming

**Figure 3:**
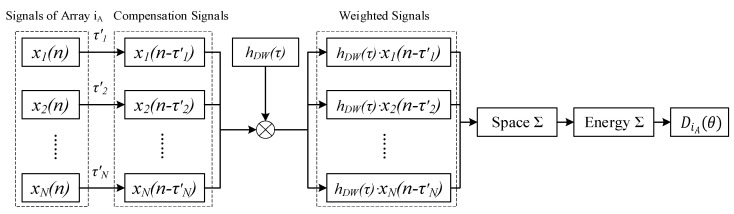
Weighted beamforming.

The weighted beamforming process of a single array after obtaining the $h_{DW}\left(\tau \right)$ is shown in Figure 3. First, the compensation time delay ${\tau^{\prime }}_{i}$ (i = 1, 2……*N*) is performed on the signals $x_{i}\left(t\right)$ received by the array $i_{A}$ ($i_{A}$ = 1, 2……*M*) to obtain the compensation signals $x_{i}\left(t-{\tau^{\prime }}_{i}\right)$. Then, the weighted signals $h_{DW}\left(\tau \right)x_{i}\left(t-{\tau^{\prime }}_{i}\right)$ are calculated by multiplying $x_{i}\left(t-{\tau^{\prime }}_{i}\right)$ and $h_{DW}\left(\tau \right)$. Subsequently, the spatial integration of $h_{DW}\left(\tau \right)x_{i}\left(t-{\tau^{\prime }}_{i}\right)$ at the same instant is performed, and the time integration of all signals at different times is operated. Finally, $D_{i_{A}}\left(\theta \right)$ (beam output of array $i_{A}$) is calculated. Since $h_{DW}\left(\tau \right)$ contains spatial information, it can enhance the incoming wave direction and suppress the non-incoming wave direction, which can improve the directional ability of $D_{i_{A}}\left(\theta \right)$.

$D_{i_{A}}\left(\theta \right)$ can be expressed as:

(9) $$D_{i_{A}}(\theta )=\sum {\left|h^{T}y(n,\tau )\right|}^{2}$$

where $h^{T}=h_{DW}(\tau )[\begin{array}{cccc} 1 & 1 & \ldots & 1 \end{array}]$, $\tau =\frac{d_{1}\sin\theta }{c}$ and c is the speed of sound in the environment. When θ = $\theta_{0},{\;D}_{i_{A}}\left(\theta \right)$ has a maximum value; at this time, $\theta_{0}$ is the direction of the sound source.

The frequency domain expression can be written as:

(10) $$D_{i_{A}}(\theta )=\frac{1}{2\pi }\prod_{j=1}^{N}\prod_{i=1}^{j}\int_{-\pi }^{\pi }\rho_{ij}(\tau_{i},\tau_{j})X_{i}(e^{j\omega })X_{j}{}^{*}(e^{j\omega })e^{j\omega (\tau_{i}-\tau_{j})}d\omega$$

where $X_{i}\left(e^{j\omega }\right)$ and $X_{j}\left(e^{j\omega }\right)$ represent the Fourier transforms of the signals $x_{i}\left(t\right)$ and $x_{j}\left(t\right)$, respectively.

As the number of arrays is M, the coordinates (x, y) of the sound source can be obtained by the following equation:

(11) $$\begin{array}{l} D_{all}=\sum_{i_{A}=1}^{M}D_{i_{A}}(f_{i_{A}}(x,y))\\ (x,y)=\underset{\left(x,y\right)}{\arg\max}D_{all} \end{array}$$

where ${\;f}_{i_{A}}\left(x,\;y\right)=$ $\theta_{i_{A}}$ represents the direction of the source coordinate (x, y) relative to the normal direction of the array $i_{A}$. $f_{i_{A}}\left(x,\;y\right)$ represents a mapping from coordinate space to direction space. When $D_{all}$ is maximized, the corresponding $\left(x_{0},{\;y}_{0}\right)$ is the maximum probability of the sound source.

## 3. Simulation Analysis

**Figure 4:**
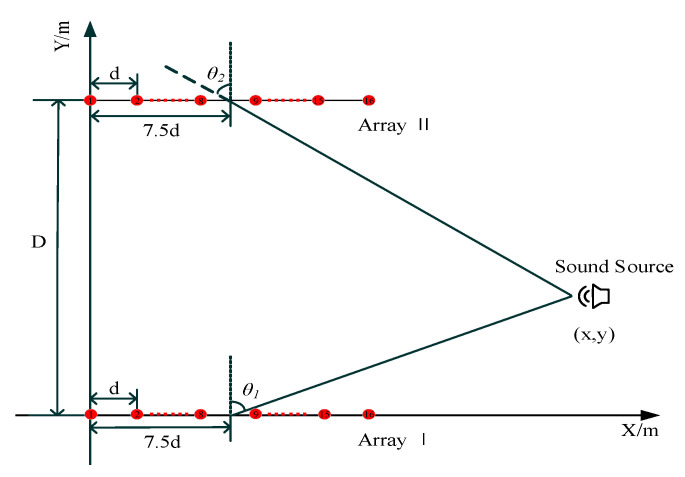
Sound source and microphone array simulation location.

We used two sets of equally spaced linear arrays to simulate the sound source localization. The coordinates of the *i*-th microphone of the array I are ((*i* − 1) × d, 0) (*i* = 1, 2, 3...16), and the coordinates of the *i*-th microphone of the array II are ((*i* − 1) × d, 10). d is the distance between two adjacent microphones and d = 0.043 m. D is the distance between the array I and array II angle and D = 10 m. The sound source and array locations are shown in Figure 4. $\theta_{1}$ and $\theta_{2}$ are sound source directions relative to array I and array II, respectively. Assuming that the normal array is the beginning edge and the incoming wave direction is the end edge, $\theta_{1}$ and $\theta_{2}$ are positive counterclockwise and are negative clockwise. Moreover, $\theta_{1}$ and $\theta_{2}\;\in [-90{}^{\circ},\;90{}^{\circ})$. The center of the array is used as a reference point, and the sound source coordinates (x, y) can be calculated by the following:

(12) $$\begin{array}{l} \frac{y}{x-7.5d}=\tan(\theta_{1}+90{}^{\circ})\\ \frac{y-D}{x-7.5d}=\tan(\theta_{2}-90{}^{\circ}) \end{array}$$

**Figure 5:**
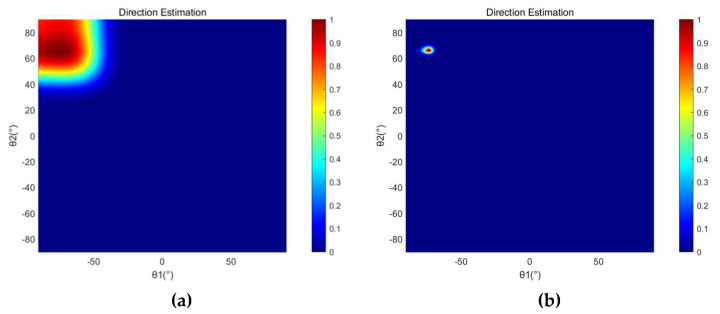
The direction estimation simulation results of (**a**) SRP-PHAT and (**b**) SRP-MCCC when the SNR is −5 dB.

**Figure 6:**
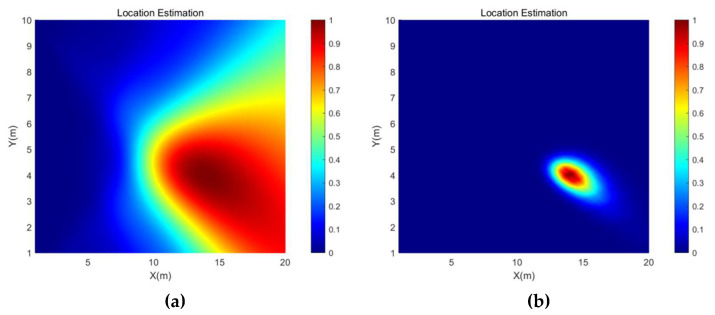
The location estimation simulation results of (**a**) SRP-PHAT and (**b**) SRP-MCCC when the SNR is −5 dB.

Figure 5 shows the direction estimation results of the SRP-PHAT and the SRP-MCCC when the source signal is a 600 Hz sine signal with source coordinates (14, 4) and the SNR is −5 dB. The brightness of a point represents the probability that the point is the location of the sound source. Figure 6 shows the location estimation results of the SRP-PHAT and the SRP-MCCC when the SNR is −5 dB.

From Figure 5 and Figure 6, we can observe that the SRP-MCCC has a superior localization convergence capability and can improve the localization accuracy by aggregating the localization results into smaller areas and improving spatial pointing. To further explore the advantages of this method, simulation analyses of SRP-PHAT and the SRP-MCCC were conducted under different SNR conditions, and the results are shown in Table 1.

As shown in Table 1, when the SNR is high, the localization errors of both methods are comparatively insignificant. As the SNR decreases, the localization error of the SRP-PHAT increases significantly, while that of the SRP-MCCC remains at a low level. When the SNR is reduced from 10 dB to −20 dB, the position error of the SRP-PHAT increases from 1.77% to 14.71%, and the error of the SRP-MCCC only increases from 0.27% to 4.30%. Simulation experiments show that the SRP-MCCC is feasible. The localization effect is excellent, and the robustness is outstanding.

To further explore the method proposed in this paper, the single-frequency signal at different source positions and the mixed-frequency signals at the same coordinate were simulated when SNR = −10 dB. The results are shown in Table 2 and Table 3, respectively. We can infer that the average error of the single-frequency signal at different coordinates decreased by 5.69%, and that of the mixed-frequency signals decreased by 5.77% at the same coordinate.

## 4. Experiment

**Figure 7:**
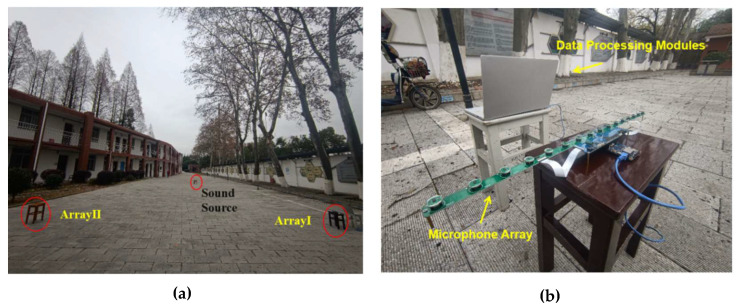
(**a**) Experimental scene. (**b**) Experimental equipment.

To verify the feasibility of the SRP-MCCC, a field localization experiment was conducted, as shown in Figure 7. The array structure and the sound source frequency are consistent with the simulation during the experiment, as shown in Figure 4. This paper presents an experimental comparison between the SRP-PHAT and the SRP-MCCC.

**Figure 8:**
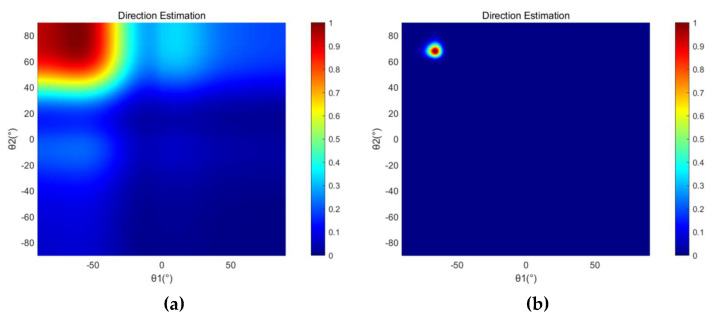
The direction estimation experimental results of (**a**) SRP-PHAT and (**b**) SRP-MCCC when the sound source coordinate is (10, 5).

**Figure 9:**
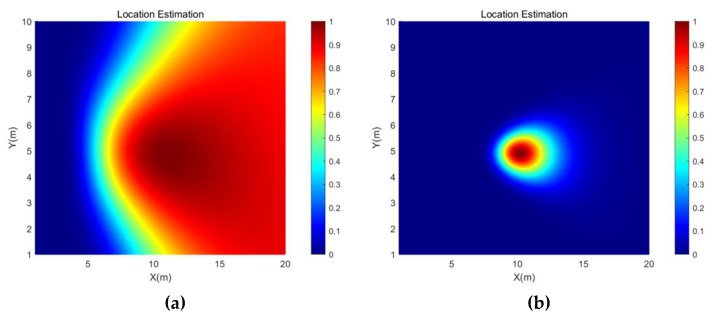
The location estimation experimental results of (**a**) SRP-PHAT and (**b**) SRP-MCCC when the sound source coordinate is (10, 5).

The direction estimation and position estimation results of SRP-PHAT and SRP-MCCC when the sound source coordinate is (10, 5) are shown in Figure 8 and Figure 9, respectively. From Figure 8 and Figure 9, it can be found that the spot size of the SRP-MCCC, in both angle and position, is smaller than SRP-PHAT. Therefore, the SRP-MCCC has a stronger angular resolution and can control the beam width in a smaller range, which can lead to more accurate direction and position estimation.

Subsequently, localization experiments were conducted for the sound source coordinates of (11, 2) and (12, 7.5), and the experimental results are shown in Table 4. Compared with the SRP-PHAT, the SRP-MCCC has higher accuracy in both direction and position estimation, and the average error is reduced by 8.14%. Therefore, SRP-MCCC has a better localization effect, which is in line with the expected results and verifies the feasibility and superiority of the SRP-MCCC.

## 5. Conclusions

A new high-precision beamforming algorithm (SRP-MCCC) is proposed to improve the positioning accuracy by combining SPR and MCCC. A detailed theoretical analysis of the method is presented here, and the simulations and experiments verify its feasibility. The results show that the method has the advantages of strong robustness and high localization accuracy. Furthermore, the SRP-MCCC has better spatial resolution and localization capability than the SRP-PHAT. Both the simulations and experiments verify the effectiveness of the method. These results provide a new idea for the weighted beamforming algorithm, which is essential for researching high-precision sound source localization in complex environments.

## Figures and Tables

**Table 1:** Simulation results for different SNR conditions (600 Hz, (14, 4)).

SNR/dB	SRP-PHAT			SRP-MCCC		
	Position/m	Directional Deviation/°	Distance Error/%	Position/m	Directional Deviation/°	Distance Error/%
10	(14.25, 3.94)	(−0.36, 0.12)	1.77	(13.96, 4.00)	(0.04, −0.08)	0.27
0	(14.27, 3.86)	(−0.46, −0.28)	2.09	(14.19, 3.96)	(−0.26, −0.03)	1.33
−5	(15.24, 3.84)	(−1.76, 1.12)	8.59	(13.78, 3.90)	(−0.11, −0.83)	1.66
−10	(12.59, 4.10)	(1.59, −2.03)	9.71	(14.31, 4.13)	(0.24, 0.87)	2.31
−15	(15.61, 4.06)	(−1.33, 2.29)	11.06	(13.48, 4.10)	(1.01, −0.46)	3.64
−20	(16.14, 4.07)	(−1.76, 3.02)	14.71	(14.57, 4.26)	(0.49, 1.57)	4.30

**Table 2:** Simulation results for different source locations (SNR = −10 dB, 600 Hz).

Source Location/m	SRP-PHAT			SRP-MCCC		
	Position/m	Directional Deviation/°	Distance Error/%	Position/m	Directional Deviation/°	Distance Error/%
(12, 7)	(10.96, 6.62)	(1.28, −3.53)	8.81	(12.22, 6.79)	(−0.96, −0.84)	2.19
(15, 6)	(16.26, 6.18)	(1.41, 1.11)	7.88	(15.52, 6.03)	(−0.46, 0.51)	3.22
(17, 4.5)	(18.27, 4.04)	(−2.34, −0.23)	7.51	(17.47, 4.60)	(−0.02, 0.85)	2.73
(19, 6.5)	(20.99, 6.84)	(0.78, 1.87)	10.05	(19.81, 6.92)	(0.46, 1.58)	4.05
(21, 5)	(21.21, 4.15)	(1.08, −0.63)	8.83	(21.52, 4.90)	(−0.62, 0.50)	2.45

**Table 3:** Simulation results for different frequencies (SNR = −10 dB, (15, 5)).

Frequency/Hz	SRP-PHAT			SRP-MCCC		
	Position/m	Directional Deviation/°	Distance Error/%	Position/m	Directional Deviation/°	Distance Error/%
600	(13.59, 5.09)	(2.21, −1.52)	8.93	(14.61, 5.09)	(0.81, −0.16)	2.53
600, 900	(16.17, 5.21)	(−0.62, 2.00)	7.52	(15.41, 5.13)	(−0.03, 0.92)	2.72
600, 900, 1500	(13.68, 5.08)	(2.03, −1.42)	8.36	(14.73, 5.23)	(1.15, 0.50)	2.24

**Table 4:** Localization results of SRP-PHAT and MCCC-SRP at different coordinates.

Source Location/m	$(\theta_{1},{\;\theta }_{2})/\text{°}$	SRP-PHAT			SRP-MCCC		
		Direction/°	Position/m	Error/%	Direction/°	Position/m	Error/%
(10, 5)	(−64.16, 64.16)	(−65.60, 66.08)	(11.14, 4.80)	10.35	(−64.99, 64.18)	(10.20, 4.91)	1.96
(11, 2)	(−79.98, 54.76)	(−81.94, 55.73)	(11.83, 1.72)	7.83	(−79.11, 54.75)	(10.81, 2.13)	2.06
(12, 7.5)	(−58.67, 78.53)	(−61.77, 79.12)	(13.39, 7.36)	12.62	(−59.52, 78.26)	(12.24, 7.39)	2.36

## Data Availability

The data used to support the findings of this study are available from the corresponding author upon request.
